# Targeting cancer stem cells *via* integrin β4

**DOI:** 10.18632/oncotarget.27977

**Published:** 2021-08-31

**Authors:** Hannah E. Dobson, Shasha Ruan, Alfred E. Chang, Max S. Wicha, Qiao Li

**Affiliations:** ^1^Rogel Cancer Center, University of Michigan, Ann Arbor, MI 48109, USA; ^2^Department of Clinical Oncology, Renmin Hospital of Wuhan University, Wuhan, Hubei 430060, China

**Keywords:** cancer stem cells, ITGβ4, dendritic cell vaccine, bispecific antibody, T cells

## Abstract

Integrins mediate cell-cell interactions and communication with the extracellular matrix (ECM). These transmembrane protein receptors allow binding between a cell and its surroundings, initiating a breadth of intracellular signaling resulting in proliferation, differentiation, survival, or migration. Such responses have made integrins an attractive target for cancer therapy. Self-renewing and highly tumorigenic cancer stem cells (CSCs) are most resistant to traditional radiation treatment and chemotherapy, and therefore may contribute directly to the metastasis and relapse of the disease. In both the 4T1 mouse metastatic mammary tumor model and SCC7 head and neck squamous cell carcinoma model, integrin β4 (ITGB4) was expressed on ALDH^high^ 4T1 and SCC7 CSCs. Using two immunological approaches, we targeted ITGB4 through 1) ITGB4 protein-pulsed dendritic cell (ITGB4-DC) vaccination or 2) via anti-CD3/anit-ITGB4 bispecific antibody (ITGB4 BiAb)-armed T cell adoptive transfer. These two therapies reduced ITGB4-expressing CSCs and inhibited local tumor growth and lung metastasis through ITGB4 specific cellular and humoral immune responses. Additionally, the combination of anti-PD-L1 immunotherapy with our two ITGB4-targeted approaches significantly improved treatment efficacy. We also found increased concentrations of serum IFN-γ and IL-6 in the 4T1 and SCC7 models which may help define future directions of this ITGB4-targeted study. Together, these results emphasize ITGB4 as a practical CSC immunological target with possible therapeutic benefits across tumor types with high ITGB4 expression.

## INTRODUCTION

The main intermediaries between a cell and its extracellular environment are integrins. Integrins are αβ heterodimers found on the surface of all nucleated cells. Each integrin subunit contains a cytoplasmic tail and one short transmembrane cross, with the remainder of the protein located outside the cell [1]. Humans have 18 α and 8 β subunits which form pairs in the endoplasmic reticulum to produce 24 distinct receptors with nonredundant function [2]. At the cell surface, ligand binding occurs at the extracellular domain formed by the meeting of the α and β subunits. Inside the cell, an integrin’s cytoplasmic tail is naturally short, incorporating only 30–50 amino acids, except for the β4 subunit which contains a cytoplasmic tail about 20 times longer (1000 amino acids) than average [3]. Subclasses of integrins include collagen receptors, leukocyte-specific receptors, laminin receptors, and RGD peptide motifs [4].

As cell adhesion molecules, integrins function as transmembrane bridges capable of communicating bidirectional signals though initiation of signaling pathways. Typically integrins exist in three states: inactive, and low-affinity or high-affinity active; these conformations impact ligand binding and also subsequent intracellular signaling [5]. Once an integrin binds its ligand, changes to the receptor’s cytoplasmic tails occur, promoting recruitment of adaptor proteins, scaffolding, and tyrosine kinases; the culmination of this process is often called the adhesome [6–8]. This complex fosters activation of signaling cascades responsible for mediating an array of cellular processes involving differentiation, migration, and survival [7, 8]. More specifically, the laminin receptor, α6β4 consisting of integrin β4 (ITGB4) dimerized with integrin α6 in epithelial cells, helps maintain the structural integrity of epithelial monolayers at the basal surface adjacent to the basement membrane [9]. The consequences of irregular or aberrant α6β4 integrin signaling can be severe. ITGB4 gene mutations can cause lethal skin and mucosal tissue disorders resulting in blistering and ulceration, and ITGB4 knockout mice die shortly after birth showing acute epidermal blistering and separation of the epithelial-mesenchymal junction [8–11].

## RESEARCH PERSPECTIVE

Integrin α6β4’s role in the maintenance of the epithelial-mesenchymal junction has implicated atypical expression patterns of this integrin complex in several malignancies including breast, bladder, cervical, head and neck, and lung cancer [9, 12]. Within the tumor microenvironment, exploitation of integrin-mediated signals encourages cancer invasion and survival [9, 13]. Several studies have linked integrin α6β4 to PI3K activation, a signaling pathway well known to encourage cancer progression [9, 14–17]. Crosstalk between the α6β4 integrin and cytokines, growth factors or spatial changes in the ECM also contributes to its role in both tumorigenesis and angiogenesis [9, 18–20]. For example, deletion of the ITGB4 signaling domain in a mouse model of ErbB2-induced mammary carcinoma resulted in suppression of tumor growth and metastasis, and improved efficacy of ErbB2-targeted therapy [21].

Some integrins have been found to regulate cell stemness [13, 19]. This finding is particularly interesting in the context of cancer stem cells (CSCs), a highly tumorigenic, self-renewing tumor cell sub-population resistant to traditional radiation and chemotherapy [22]. Yoshioka *et al*. traced deletion of ITGB4 back to defective signaling of ErB2 and c-Met in prostate tumor progenitor cells; inhibition of ErbB2 and c-Met subsequently reduced the ability of tumor progenitor cells to self-renew *in vitro*, suggesting ITGB4 signaling may play a critical role in maintaining CSC within the tumor [23, 24]. More recently, Bierie and colleagues used ITGB4 as a functional biomarker to classify more aggressive CSCs from other mesenchymal carcinoma cell subsets in triple-negative breast cancer [25]. The implications of ITGB4’s oncogenic potential and contribution to CSCs make this integrin subclass an appealing immunological target for cancer treatment.

**Figure 1:**
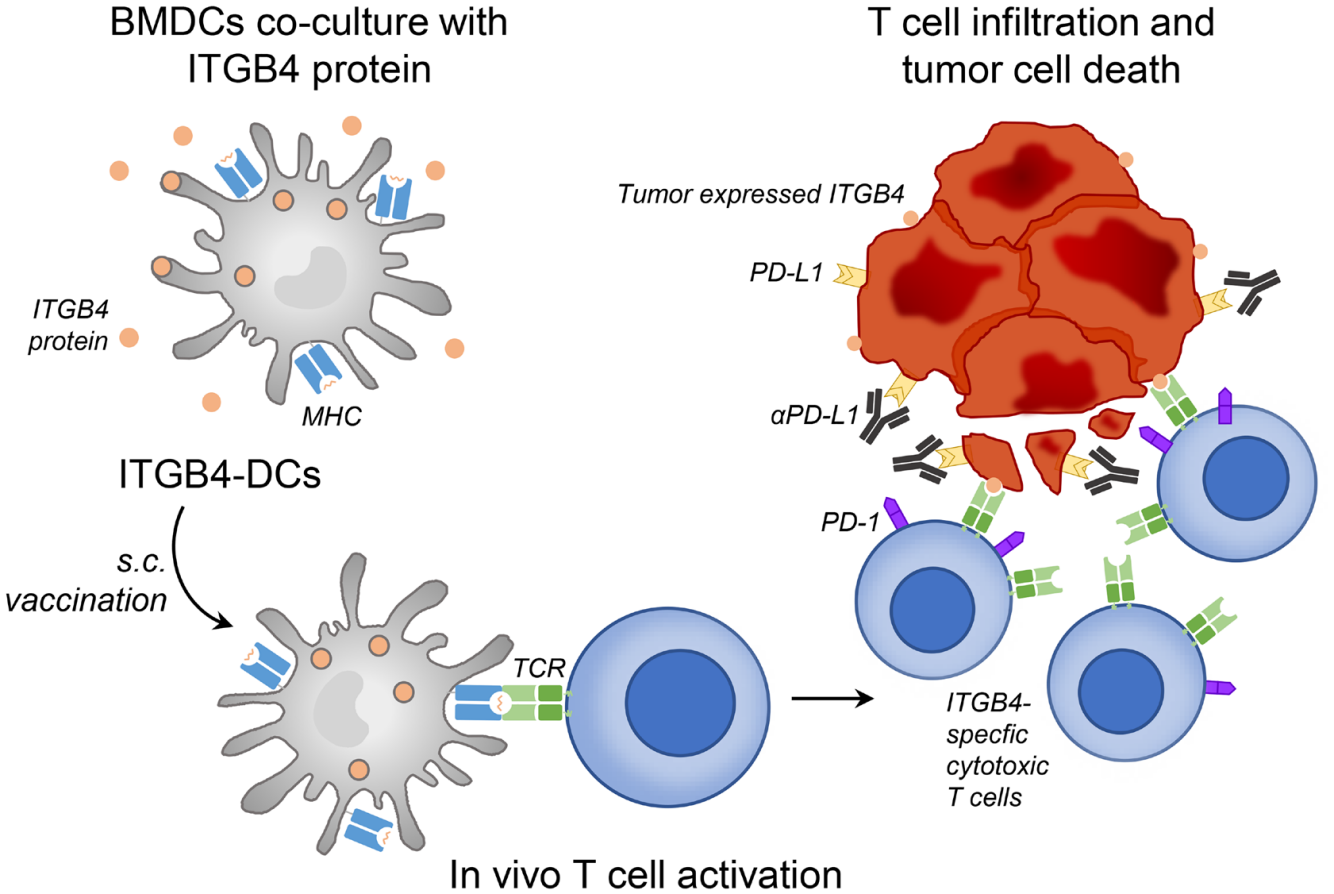
ITGB4-DC vaccine primes ITGB4-specific T cells *in vivo*, leading to killing of ITGB4 expressing cancer stem cells and differentiated tumor cells which is augmented by anti-PD-L1 administration. Murine bone marrow derived DCs were isolated and expanded with GM-CSF and 2-mercaptoethanol. Approximately 1 × 10^6^ activated DCs were cultured with 20 μg of ITGB4 protein overnight to generate ITGB4-DC vaccines. Each vaccine dose contained 2 × 10^6^ ITGB4 pulsed DCs and was delivered subcutaneously.

**Figure 2:**
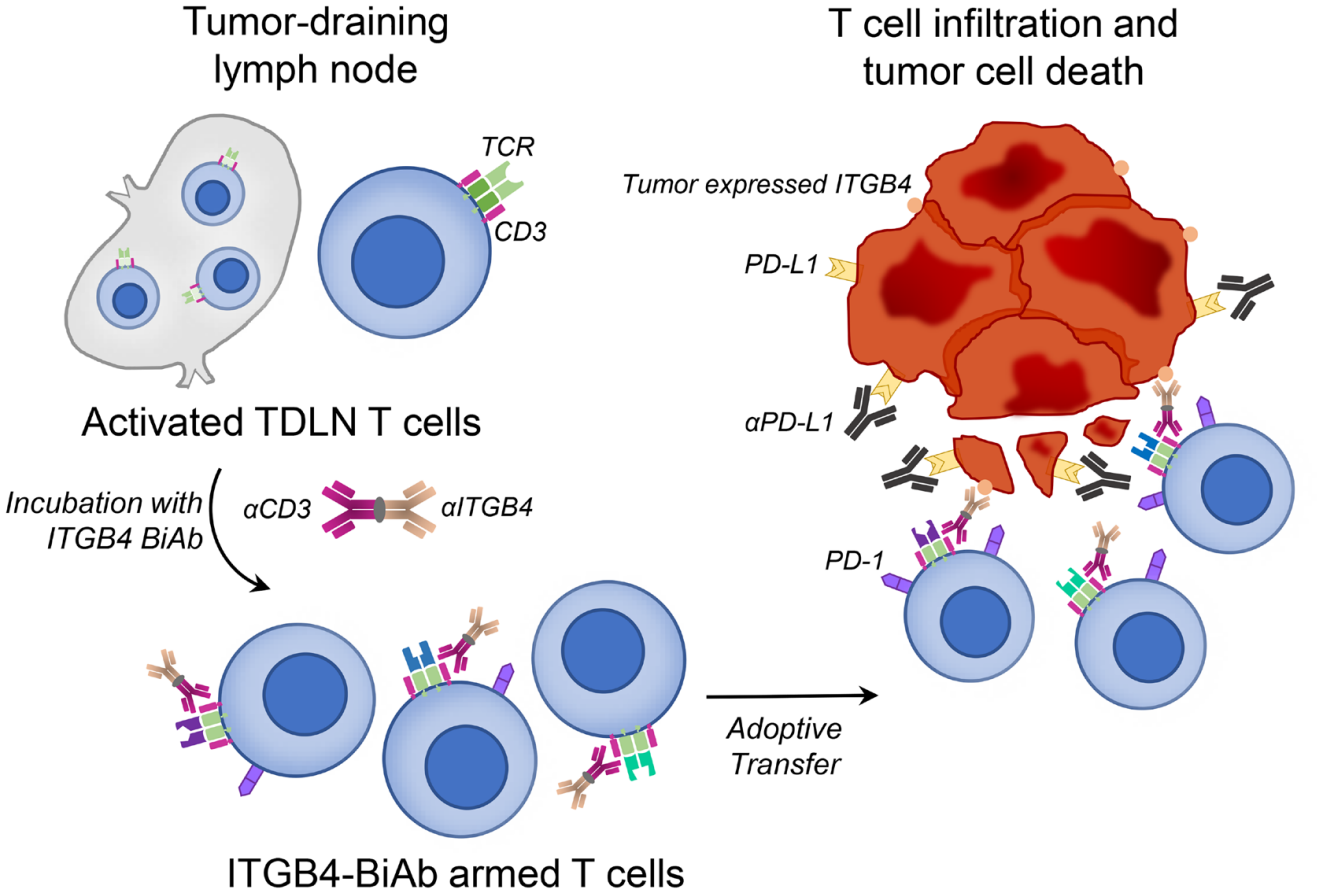
Adoptive transfer of ITGB4-BiAb armed T cells, combined with αPD-L1 administration, results in killing of ITGB4 expressing cancer stem cells and differentiated tumor cells. Tumor draining lymph nodes (TDLN) T cells were harvested and activated/expanded *in vitro* with αCD3, αCD28, and IL-2. Activated and expanded TDLN T cells were then incubated with anti-CD3/anti-ITGB4 BiAb to generate ITGB4-BiAb armed T cells. These T cells were adoptively transferred into tumor-bearing mice for immunotherapy.

These findings, linking ITGB4 expression and CSCs, led our group to investigate the therapeutic efficacy of targeting CSCs *via* ITGB4. We applied two distinct targeting approaches: 1) vaccination with ITGB4 protein-pulsed dendritic cells (ITGB4-DCs) and 2) adoptive transfer of anti-CD3/anti-ITGB4 bispecific antibody (ITGB4 BiAb)-armed T cells [26]. We and others have demonstrated the effectiveness of DC-based cancer vaccines in melanoma, head and neck, and other murine tumor cell models [27–29] as well as in clinical trials [30, 31]. In our previous reports, we used aldehyde dehydrogenase (ALDH), a widely accepted CSC marker, to isolate ALDH^high^ CSCs and generate CSC lysates. Subsequently we pulsed this lysate with DCs to produce CSC lysate-DC vaccines, which effectively targeted ALDH^high^ CSCs [27, 28, 32–34]. However, this method is clinically limited by the availability of patient tumor sample, a requirement for the isolation of ALDH^high^ CSCs. With ITGB4 expression found on several tumor types [23, 25, 35, 36], immunological targeting of CSCs through ITGB4 protein-pulsed DC vaccination provides a more accessible route to clinical relevance (Figure 1) [26]. In our second approach, we generated an anti-CD3/anti-ITGB4 bispecific antibody and bound it to activated and expanded tumor draining lymph node (TDLN) T cells (Figure 2). Bispecific antibodies can elicit a diverse effector T cell population through CD3 binding, and guide these cytotoxic effectors towards a specific tumor antigen, e.g. ITGB4. Currently, there are two FDA approved bispecific antibodies for clinical use: blinatumomab, used to treat acute hematological malignancies via CD3 and CD19, and emicizumab, which binds the coagulation factor IX and factor X for treatment of hemophilia A [37]. Several other bispecific antibodies are undergoing pre-clinical and clinical trials to treat lung, colorectal, prostate, breast, and ovarian cancers [38].

Both DC vaccination and bispecific antibody-directed T cell transfer rely on engagement of the host immune system. DC vaccination requires effective antigen presentation by MHC followed by activation and migration of T lymphocytes to drive cytotoxic anti-tumor immunity, while bispecific antibodies rely on bound T cells to reach and interact with the intended tumor antigen [29, 38]. Pitfalls in these approaches often lie in the immunosuppressive tumor microenvironment, where the potentially productive anti-tumor immunity induced by such therapies can be inhibited [39]. Tumors employ several methods which contribute to host immune suppression, e.g. via oncogenic cytokine production driving immunosuppressive cell subsets to PD-L1 self-expression dampening the effector function of PD-1^+^ cytotoxic lymphocytes [39]. To reduce immunosuppression and encourage effective immune responses, checkpoint blockade using anti-PD-1/PD-L1 antibodies has become standard therapy for several cancers [39, 40]. Therefore, in addition to assessing mono-therapeutic benefit of our two ITGB4-targeted approaches, we also combined our ITGB4-DC vaccination and ITGB4-BiAb armed T cell transfer with anti-PD-L1 immunotherapy (Figures 1 and 2) [26].

The 4T1 and SCC7 murine tumor cells expressed ITGB4 and PD-L1 on bulk tumor cells and ALDH^high^ CSCs, making both proteins viable immunological targets [26]. Assessing the efficacy of our ITGB4-DC vaccine in these two tumor models, we found that vaccination combined with anti-PD-L1 immunotherapy significantly reduced tumor growth and inhibited spontaneous pulmonary metastasis better than each monotherapy. Similarly, we observed diminished tumor growth and metastasis in established tumors after adoptive transfer of ITGB4-BiAb armed TDLN cells plus anti-PD-L1 administration [26].

To validate ITGB4 targeting, we generated 4T1-ITGB4^KO^ tumor cells and evaluated the specificity of our two ITGB4-directed treatment protocols. Remarkably, treatment with ITGB4-DC vaccine or ITGB4-BiAb TDLN T cells did not improve 4T1-ITGB4^KO^ tumor growth, strongly suggesting that both of our ITGB4-targeted immunological strategies were ITGB4 specific. These findings were further validated in an ITGB4^low^ cell line, CT26. Additionally, 4T1-ITGB4^KO^ tumors showed improved growth responses compared to both untreated and treated WT-4T1 tumors, implying that ITGB4 signaling participates in tumor development within the 4T1 model [26].

Functionally, we established via cytotoxicity, serum binding, and complement assays that ITGB4-DC vaccination, combined with anti-PD-L1 immunotherapy, induced cytotoxic splenic T cells and serum antibodies capable of mediating the binding and killing of both bulk tumor cells and ALDH^high^ CSCs. *In vitro* functional responses against CSCs were confirmed by a reduction in number and tumorigenicity of ITGB4^+^ ALDH^high^ CSCs in residual tumors after treatment, most significantly after combination therapy [26]. Together, these findings support the use of ITGB4-targeted treatment, combined with anti-PD-L1 immunotherapy, as a method to specifically target ITGB4-expressing tumor cells and CSCs.

### Modulation of IFN-γ and IL-6 after ITGB4-targeted therapy

**Figure 3:**
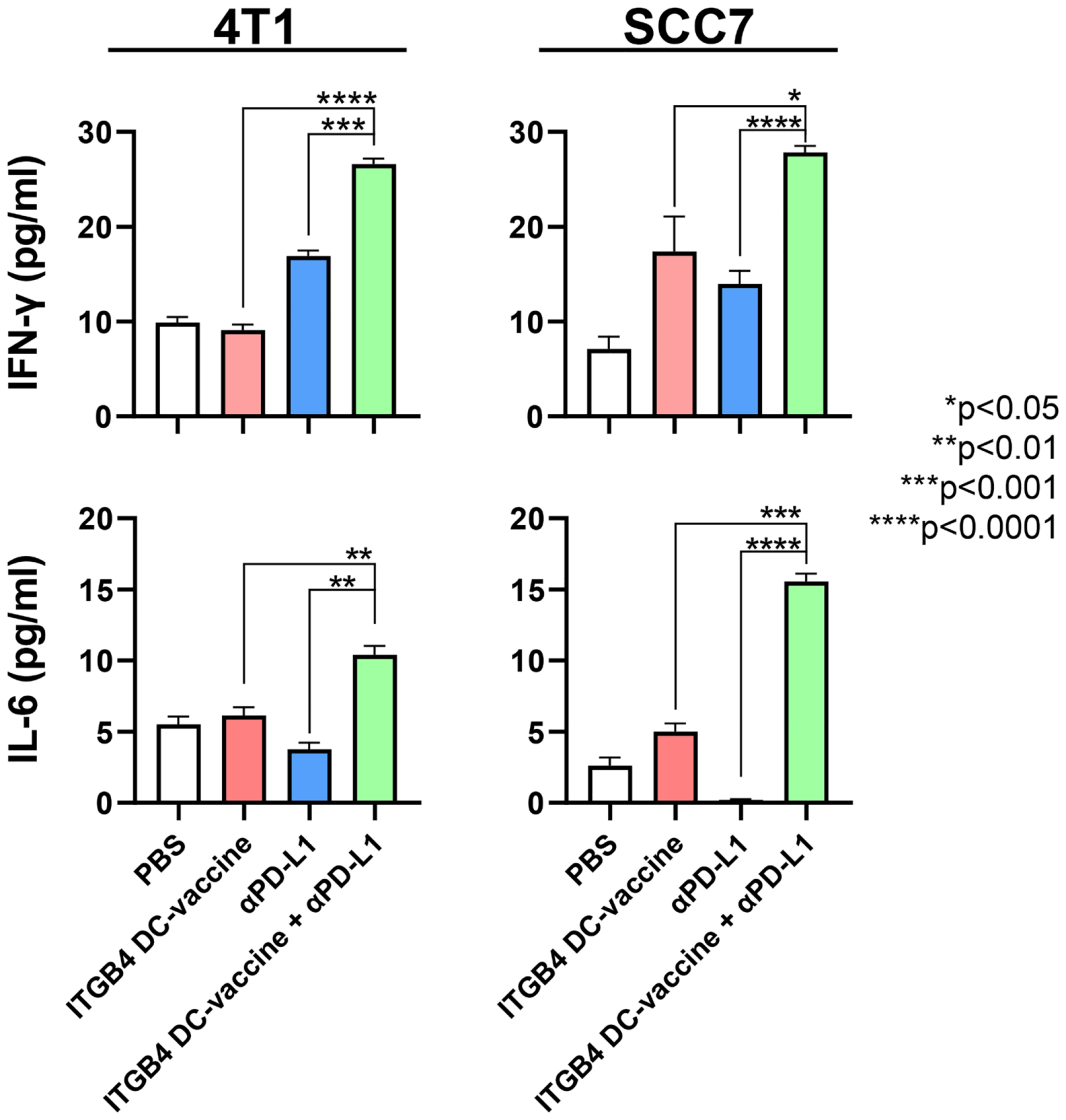
Serum concentrations of IFN-γ and IL-6 are significantly increased following vaccination with ITGB4-DCs combined with anti-PD-L1 administration. Serum samples were collected from tumor bearing mice subjected to treatment as indicated. An unpaired two-tailed *t*-test was used to calculate *p*-values between combination therapy and monotherapies.

Of note, after an observable reduction in tumor growth and metastasis following ITGB4-DC vaccination plus anti-PD-L1 immunotherapy, we found increased serum levels of IFN-γ and IL-6 compared to controls in both the 4T1 and SCC7 models (Figure 3). IFN-γ and IL-6 play dynamic and complex roles in malignant diseases. CD8 and CD4 T cell produce IFN-γ during the adaptive immune response which favors Th1 cell expansion, and expression of IFN-γ has been shown to upregulate MHC class I on tumor cells and increase cytotoxicity of natural killer cells and cytotoxic T cells [41, 42]. IFN-γ can also induce apoptosis of tumor cells and thus augment anti-tumor effects of immunotherapy [43–45]. However, a recent study by Song *et al.* suggests that low levels of IFN-γ contribute to tumor cell stemness and metastasis, while high dose IFN-γ resulted in tumor regression in non-small cell lung cancer [43].

Like IFN-γ, IL-6 also influences several important biological functions, including regulation of lymphocyte differentiation and chronic inflammation [44]. IL-6 impacts the balance between Th1 and Th2 effector responses and has been shown to inhibit TGF-β-induced Foxp3^+^ T_reg_ cell formation [45, 46]. Additionally, IL-6 aids in the early differentiation of T follicular helper cells, a subset of lymphocytes which facilitate the formation of germinal centers, memory B cells, long-lived plasma cells, and affinity maturation [47]. Two signaling pathways exist for IL-6: classical and trans [48]. Classical signaling occurs largely in leukocytes and liver cells and induces anti-inflammatory and regenerative responses, while trans signaling triggers pro-inflammatory pathways resulting in inflammation [48].

Reported pleiotropic activity of IFN-γ and IL-6 has also unveiled dysregulation of these cytokines in the pathogenesis of cancer [47, 49]. For example, investigators have observed PD-L1 expression on tumor cells driven by chronic low-dose exposure to IFN-γ, allowing immune escape [49–53]. Additionally, IFN-γ can contribute to metastasis through induction of CXCR4 on tumor cells and promotion of epithelial-to-mesenchymal transition [49, 54]. In regards to IL-6, with unchecked inflammation being a hallmark of the tumor microenvironment, elevated levels of this cytokine can be found in tumor tissues of numerous cancers which have linked IL-6 to aggressive tumor growth and metastasis [48]. For example, high patient serum levels of IL-6 have been correlated with poor prognosis and shorter survival [55–57]. Within the tumor microenvironment, studies have associated IL-6 production with CSCs. Krishnamurthy *et al.* determined IL-6 secreted by endothelial cells, in human head and neck squamous cell carcinoma (HNSCC), increased the tumorigenicity and survival of CSCs in athymic IL-6 knockout mice [58]. Subsequent blocking of IL-6 with the humanized anti-IL-6 antibody MEDI5117 after transplantation of HNSCC patient-derived xenografts into SCID mice successfully reduced CSCs within tumors [59].

In the context of these findings, our results in Figure 3 present a noteworthy observation of a potential synergistic relationship between IFN-γ and IL-6 during cancer therapy. IL-6 deficient mice have been reported to exhibit reduced tumor growth and metastasis, and increased IFN-γ producing CD8 T cells; despite improving tumorigenesis, IL-6^-/-^ mice showed enhanced PD-L1 expression on murine CT26 colon cancer cells which could be mitigated by anti-PD-L1 immunotherapy [60, 61]. Although the absence of IL-6 improved anti-tumor immune function, this response is potentially hampered by enhanced PD-L1 expression on tumor cells. Furthermore, Hsiao and colleagues determined that exogenous combination IFN-γ and IL-6 therapy could increase the expression of MHC class I and II molecules on tumor cells, potentially enhancing the ability of T cells to recognize and target cancer cells [62]. Therefore, a balanced IFN-γ and IL-6 response mediated by favorable cell subsets within the tumor microenvironment could be beneficial during cancer treatment, though this outcome is most likely dependent on multiple factors including cancer type, immune status, tumor stage, etc. With a significant increase in serum concentration of these two cytokines after ITGB4-DC vaccination and anti-PD-L1 immunotherapy (Figure 3), investigating their roles and function to improve ITGB4-targeted therapies warrants further investigation. Future experiments involving the administration of blocking antibodies to assess individual contribution to tumor growth, and evaluation of cytokine concentrations and lymphocyte differentiation within the tumor, might provide a better understanding of IFN-γ and IL-6 cooperation after ITGB4-targeted immunotherapy and checkpoint blockade.

### Toxicity

Toxicity following immunotherapy is a major hurdle in cancer treatment. Engaging a widely expressed integrin, such as ITGB4, in combination with anti-PD-L1 immunotherapy, risks inducing off-target effects. To evaluate the possible toxicity of ITGB4-targeting combined with anti-PD-L1, we assessed histopathology of several internal organs after treatment including lung, spleen, skin, salivary gland, esophagus, heart, kidney, and liver, and found similar inflammatory profiles among all treatment and control groups. Furthermore, we observed no difference in plasma levels of IGF-1 or hematopoiesis after either ITGB4-DC vaccination or ITGB4-BiAb T cell adoptive transfer. Together, these results suggest a lack of toxicity following ITGB4-targeted therapies and potentially demonstrate translatable safety.

### Concluding remarks

As an invasive and malignant disease, cancer manipulates normal biology for growth and survival. In healthy tissue, integrins allow bidirectional communication between a cell and its extracellular environment. Given their critical role as environmental sensors, atypical integrin expression by tumor cells can promote tumorigenesis, local invasion, and metastasis through crosstalk between cytokines and growth factors and undesirable cell adhesion to the ECM. Integrin signaling can also mediate cell stemness and potentially contribute to the renewal of highly tumorigenic CSCs. We have shown in the 4T1 and SCC7 mouse models that both unsorted tumor cells and ALDH^high^ CSCs express ITGB4, and immunological targeting of ITGB4 combined with anti-PD-L1 immunotherapy elicits significant anti-tumor immunity. With ITGB4 overexpression found across several cancer types, and its association as an adverse prognostic marker, ITGB4-focused therapies represent a potential therapeutic approach to direct host immune responses against both CSCs and bulk tumor cells. Further investigation into the synergistic relationship between IFN-γ and IL-6 may provide insight into how these ITGB4-targeted immunotherapies function *in vivo*, and potentially provide additional effort to improve cancer therapeutic efficacy.

## References

[1] Hynes RO. Integrins: versatility, modulation, and signaling in cell adhesion. Cell. 1992; 69:11–25. 10.1016/0092-8674(92)90115-s. 1555235

[2] Anderson LR, Owens TW, Naylor MJ. Structural and mechanical functions of integrins. Biophys Rev. 2014; 6:203–13. 10.1007/s12551-013-0124-0. 28510180 PMC5418412

[3] van der Flier A, Sonnenberg A. Function and interactions of integrins. Cell Tissue Res. 2001; 305:285–98. 10.1007/s004410100417. 11572082

[4] Hynes RO. Integrins: bidirectional, allosteric signaling machines. Cell. 2002; 110:673–87. 10.1016/s0092-8674(02)00971-6. 12297042

[5] Shi M, Foo SY, Tan SM, Mitchell EP, Law SK, Lescar J. A structural hypothesis for the transition between bent and extended conformations of the leukocyte beta2 integrins. J Biol Chem. 2007; 282:30198–206. 10.1074/jbc.M701670200. 17673459

[6] Mezu-Ndubuisi OJ, Maheshwari A. The role of integrins in inflammation and angiogenesis. Pediatr Res. 2020 10 7. 10.1038/s41390-020-01177-9. [Epub ahead of print]. 33027803 PMC8249239

[7] Horton ER, Humphries JD, James J, Jones MC, Askari JA, Humphries MJ. The integrin adhesome network at a glance. J Cell Sci. 2016; 129:4159–63. 10.1242/jcs.192054. 27799358 PMC5117201

[8] Winograd-Katz SE, Fässler R, Geiger B, Legate KR. The integrin adhesome: from genes and proteins to human disease. Nat Rev Mol Cell Biol. 2014; 15:273–88. 10.1038/nrm3769. 24651544

[9] Stewart RL, O’Connor KL. Clinical significance of the integrin α6β4 in human malignancies. Lab Invest. 2015; 95:976–86. 10.1038/labinvest.2015.82. 26121317 PMC4554527

[10] Vidal F, Aberdam D, Miquel C, Christiano AM, Pulkkinen L, Uitto J, Ortonne JP, Meneguzzi G. Integrin beta 4 mutations associated with junctional epidermolysis bullosa with pyloric atresia. Nat Genet. 1995; 10:229–34. 10.1038/ng0695-229. 7545057

[11] Dowling J, Yu QC, Fuchs E. Beta4 integrin is required for hemidesmosome formation, cell adhesion and cell survival. J Cell Biol. 1996; 134:559–72. 10.1083/jcb.134.2.559. 8707838 PMC2120864

[12] Hamidi H, Ivaska J. Every step of the way: integrins in cancer progression and metastasis. Nat Rev Cancer. 2018; 18:533–48. 10.1038/s41568-018-0038-z. 30002479 PMC6629548

[13] Hamidi H, Pietilä M, Ivaska J. The complexity of integrins in cancer and new scopes for therapeutic targeting. Br J Cancer. 2016; 115:1017–23. 10.1038/bjc.2016.312. 27685444 PMC5117799

[14] Shaw LM, Rabinovitz I, Wang HH, Toker A, Mercurio AM. Activation of phosphoinositide 3-OH kinase by the alpha6beta4 integrin promotes carcinoma invasion. Cell. 1997; 91:949–60. 10.1016/s0092-8674(00)80486-9. 9428518

[15] Gambaletta D, Marchetti A, Benedetti L, Mercurio AM, Sacchi A, Falcioni R. Cooperative signaling between alpha(6)beta(4) integrin and ErbB-2 receptor is required to promote phosphatidylinositol 3-kinase-dependent invasion. J Biol Chem. 2000; 275:10604–10. 10.1074/jbc.275.14.10604. 10744756

[16] Gagnoux-Palacios L, Dans M, van’t Hof W, Mariotti A, Pepe A, Meneguzzi G, Resh MD, Giancotti FG. Compartmentalization of integrin alpha6beta4 signaling in lipid rafts. J Cell Biol. 2003; 162:1189–96. 10.1083/jcb.200305006. 14517202 PMC2173954

[17] Bhatia V, Mula RV, Weigel NL, Falzon M. Parathyroid hormone-related protein regulates cell survival pathways via integrin alpha6beta4-mediated activation of phosphatidylinositol 3-kinase/Akt signaling. Mol Cancer Res. 2009; 7:1119–31. 10.1158/1541-7786.MCR-08-0568. 19584267 PMC2755566

[18] Ivaska J, Heino J. Cooperation between integrins and growth factor receptors in signaling and endocytosis. Annu Rev Cell Dev Biol. 2011; 27:291–320. 10.1146/annurev-cellbio-092910-154017. 21663443

[19] Desgrosellier JS, Cheresh DA. Integrins in cancer: biological implications and therapeutic opportunities. Nat Rev Cancer. 2010; 10:9–22. 10.1038/nrc2748. 20029421 PMC4383089

[20] Avraamides CJ, Garmy-Susini B, Varner JA. Integrins in angiogenesis and lymphangiogenesis. Nat Rev Cancer. 2008; 8:604–17. 10.1038/nrc2353. 18497750 PMC2577722

[21] Guo W, Pylayeva Y, Pepe A, Yoshioka T, Muller WJ, Inghirami G, Giancotti FG. Beta 4 integrin amplifies ErbB2 signaling to promote mammary tumorigenesis. Cell. 2006; 126:489–502. 10.1016/j.cell.2006.05.047. 16901783

[22] Yu Z, Pestell TG, Lisanti MP, Pestell RG. Cancer stem cells. Int J Biochem Cell Biol. 2012; 44:2144–51. 10.1016/j.biocel.2012.08.022. 22981632 PMC3496019

[23] Yoshioka T, Otero J, Chen Y, Kim YM, Koutcher JA, Satagopan J, Reuter V, Carver B, de Stanchina E, Enomoto K, Greenberg NM, Scardino PT, Scher HI, et al. β4 Integrin signaling induces expansion of prostate tumor progenitors. J Clin Invest. 2013; 123:682–99. 10.1172/JCI60720. 23348745 PMC3561800

[24] Wicha MS. B4 androgen ablation: attacking the prostate cancer stem cell. J Clin Invest. 2013; 123:563–65. 10.1172/JCI67460. 23348735 PMC3561837

[25] Bierie B, Pierce SE, Kroeger C, Stover DG, Pattabiraman DR, Thiru P, Liu Donaher J, Reinhardt F, Chaffer CL, Keckesova Z, Weinberg RA. Integrin-β4 identifies cancer stem cell-enriched populations of partially mesenchymal carcinoma cells. Proc Natl Acad Sci U S A. 2017; 114:E2337–46. 10.1073/pnas.1618298114. 28270621 PMC5373369

[26] Ruan S, Lin M, Zhu Y, Lum L, Thakur A, Jin R, Shao W, Zhang Y, Hu Y, Huang S, Hurt EM, Chang AE, Wicha MS, Li Q. Integrin β4-Targeted Cancer Immunotherapies Inhibit Tumor Growth and Decrease Metastasis. Cancer Res. 2020; 80:771–83. 10.1158/0008-5472.CAN-19-1145. 31843981 PMC7024642

[27] Ning N, Pan Q, Zheng F, Teitz-Tennenbaum S, Egenti M, Yet J, Li M, Ginestier C, Wicha MS, Moyer JS, Prince ME, Xu Y, Zhang XL, et al. Cancer stem cell vaccination confers significant antitumor immunity. Cancer Res. 2012; 72:1853–64. 10.1158/0008-5472.CAN-11-1400. 22473314 PMC3320735

[28] Lu L, Tao H, Chang AE, Hu Y, Shu G, Chen Q, Egenti M, Owen J, Moyer JS, Prince ME, Huang S, Wicha MS, Xia JC, Li Q. Cancer stem cell vaccine inhibits metastases of primary tumors and induces humoral immune responses against cancer stem cells. Oncoimmunology. 2015; 4:e990767. 10.4161/2162402X.2014.990767. 25949905 PMC4404925

[29] Calmeiro J, Carrascal MA, Tavares AR, Ferreira DA, Gomes C, Falcão A, Cruz MT, Neves BM. Dendritic Cell Vaccines for Cancer Immunotherapy: The Role of Human Conventional Type 1 Dendritic Cells. Pharmaceutics. 2020; 12:158. 10.3390/pharmaceutics12020158. 32075343 PMC7076373

[30] Chang AE, Redman BG, Whitfield JR, Nickoloff BJ, Braun TM, Lee PP, Geiger JD, Mulé JJ. A phase I trial of tumor lysate-pulsed dendritic cells in the treatment of advanced cancer. Clin Cancer Res. 2002; 8:1021–32. 11948109

[31] Redman BG, Chang AE, Whitfield J, Esper P, Jiang G, Braun T, Roessler B, Mulé JJ. Phase Ib trial assessing autologous, tumor-pulsed dendritic cells as a vaccine administered with or without IL-2 in patients with metastatic melanoma. J Immunother. 2008; 31:591–98. 10.1097/CJI.0b013e31817fd90b. 18528294 PMC2642589

[32] Toledo-Guzmán ME, Hernández MI, Gómez-Gallegos ÁA, Ortiz-Sánchez E. ALDH as a Stem Cell Marker in Solid Tumors. Curr Stem Cell Res Ther. 2019; 14:375–88. 10.2174/1574888X13666180810120012. 30095061

[33] Visus C, Wang Y, Lozano-Leon A, Ferris RL, Silver S, Szczepanski MJ, Brand RE, Ferrone CR, Whiteside TL, Ferrone S, DeLeo AB, Wang X. Targeting ALDH(bright) human carcinoma-initiating cells with ALDH1A1-specific CD8^+^ T cells. Clin Cancer Res. 2011; 17:6174–84. 10.1158/1078-0432.CCR-11-1111. 21856769 PMC3186874

[34] Clay MR, Tabor M, Owen JH, Carey TE, Bradford CR, Wolf GT, Wicha MS, Prince ME. Single-marker identification of head and neck squamous cell carcinoma cancer stem cells with aldehyde dehydrogenase. Head Neck. 2010; 32:1195–201. 10.1002/hed.21315. 20073073 PMC2991066

[35] Hoshino A, Costa-Silva B, Shen TL, Rodrigues G, Hashimoto A, Tesic Mark M, Molina H, Kohsaka S, Di Giannatale A, Ceder S, Singh S, Williams C, Soplop N, et al. Tumour exosome integrins determine organotropic metastasis. Nature. 2015; 527:329–35. 10.1038/nature15756. 26524530 PMC4788391

[36] Kawakami K, Fujita Y, Kato T, Mizutani K, Kameyama K, Tsumoto H, Miura Y, Deguchi T, Ito M. Integrin β4 and vinculin contained in exosomes are potential markers for progression of prostate cancer associated with taxane-resistance. Int J Oncol. 2015; 47:384–90. 10.3892/ijo.2015.3011. 25997717

[37] Labrijn AF, Janmaat ML, Reichert JM, Parren PW. Bispecific antibodies: a mechanistic review of the pipeline. Nat Rev Drug Discov. 2019; 18:585–608. 10.1038/s41573-019-0028-1. 31175342

[38] Sedykh SE, Prinz VV, Buneva VN, Nevinsky GA. Bispecific antibodies: design, therapy, perspectives. Drug Des Devel Ther. 2018; 12:195–208. 10.2147/DDDT.S151282. 29403265 PMC5784585

[39] Binnewies M, Roberts EW, Kersten K, Chan V, Fearon DF, Merad M, Coussens LM, Gabrilovich DI, Ostrand-Rosenberg S, Hedrick CC, Vonderheide RH, Pittet MJ, Jain RK, et al. Understanding the tumor immune microenvironment (TIME) for effective therapy. Nat Med. 2018; 24:541–50. 10.1038/s41591-018-0014-x. 29686425 PMC5998822

[40] Wu X, Gu Z, Chen Y, Chen B, Chen W, Weng L, Liu X. Application of PD-1 Blockade in Cancer Immunotherapy. Comput Struct Biotechnol J. 2019; 17:661–74. 10.1016/j.csbj.2019.03.006. 31205619 PMC6558092

[41] Zaidi MR. The Interferon-Gamma Paradox in Cancer. J Interferon Cytokine Res. 2019; 39:30–38. 10.1089/jir.2018.0087. 30388040 PMC6350411

[42] Martini M, Testi MG, Pasetto M, Picchio MC, Innamorati G, Mazzocco M, Ugel S, Cingarlini S, Bronte V, Zanovello P, Krampera M, Mosna F, Cestari T, et al. IFN-gamma-mediated upmodulation of MHC class I expression activates tumor-specific immune response in a mouse model of prostate cancer. Vaccine. 2010; 28:3548–57. 10.1016/j.vaccine.2010.03.007. 20304037

[43] Song M, Ping Y, Zhang K, Yang L, Li F, Zhang C, Cheng S, Yue D, Maimela NR, Qu J, Liu S, Sun T, Li Z, et al. Low-Dose IFNγ Induces Tumor Cell Stemness in Tumor Microenvironment of Non-Small Cell Lung Cancer. Cancer Res. 2019; 79:3737–48. 10.1158/0008-5472.CAN-19-0596. 31085700

[44] Tanaka T, Narazaki M, Kishimoto T. IL-6 in inflammation, immunity, and disease. Cold Spring Harb Perspect Biol. 2014; 6:a016295. 10.1101/cshperspect.a016295. 25190079 PMC4176007

[45] Diehl S, Rincón M. The two faces of IL-6 on Th1/Th2 differentiation. Mol Immunol. 2002; 39:531–36. 10.1016/s0161-5890(02)00210-9. 12431386

[46] Bettelli E, Carrier Y, Gao W, Korn T, Strom TB, Oukka M, Weiner HL, Kuchroo VK. Reciprocal developmental pathways for the generation of pathogenic effector TH17 and regulatory T cells. Nature. 2006; 441:235–38. 10.1038/nature04753. 16648838

[47] Yao X, Huang J, Zhong H, Shen N, Faggioni R, Fung M, Yao Y. Targeting interleukin-6 in inflammatory autoimmune diseases and cancers. Pharmacol Ther. 2014; 141:125–39. 10.1016/j.pharmthera.2013.09.004. 24076269

[48] Kumari N, Dwarakanath BS, Das A, Bhatt AN. Role of interleukin-6 in cancer progression and therapeutic resistance. Tumour Biol. 2016; 37:11553–72. 10.1007/s13277-016-5098-7. 27260630

[49] Jorgovanovic D, Song M, Wang L, Zhang Y. Roles of IFN-γ in tumor progression and regression: a review. Biomark Res. 2020; 8:49. 10.1186/s40364-020-00228-x. 33005420 PMC7526126

[50] Abiko K, Matsumura N, Hamanishi J, Horikawa N, Murakami R, Yamaguchi K, Yoshioka Y, Baba T, Konishi I, Mandai M. IFN-γ from lymphocytes induces PD-L1 expression and promotes progression of ovarian cancer. Br J Cancer. 2015; 112:1501–09. 10.1038/bjc.2015.101. 25867264 PMC4453666

[51] Mandai M, Hamanishi J, Abiko K, Matsumura N, Baba T, Konishi I. Dual Faces of IFNγ in Cancer Progression: A Role of PD-L1 Induction in the Determination of Pro- and Antitumor Immunity. Clin Cancer Res. 2016; 22:2329–34. 10.1158/1078-0432.CCR-16-0224. 27016309

[52] Zhang X, Zeng Y, Qu Q, Zhu J, Liu Z, Ning W, Zeng H, Zhang N, Du W, Chen C, Huang JA. PD-L1 induced by IFN-γ from tumor-associated macrophages via the JAK/STAT3 and PI3K/AKT signaling pathways promoted progression of lung cancer. Int J Clin Oncol. 2017; 22:1026–33. 10.1007/s10147-017-1161-7. 28748356

[53] Bellucci R, Martin A, Bommarito D, Wang K, Hansen SH, Freeman GJ, Ritz J. Interferon-γ-induced activation of JAK1 and JAK2 suppresses tumor cell susceptibility to NK cells through upregulation of PD-L1 expression. Oncoimmunology. 2015; 4:e1008824. 10.1080/2162402X.2015.1008824. 26155422 PMC4485824

[54] Chen HC, Chou AS, Liu YC, Hsieh CH, Kang CC, Pang ST, Yeh CT, Liu HP, Liao SK. Induction of metastatic cancer stem cells from the NK/LAK-resistant floating, but not adherent, subset of the UP-LN1 carcinoma cell line by IFN-γ. Lab Invest. 2011; 91:1502–13. 10.1038/labinvest.2011.91. 21691263

[55] Shibayama O, Yoshiuchi K, Inagaki M, Matsuoka Y, Yoshikawa E, Sugawara Y, Akechi T, Wada N, Imoto S, Murakami K, Ogawa A, Akabayashi A, Uchitomi Y. Association between adjuvant regional radiotherapy and cognitive function in breast cancer patients treated with conservation therapy. Cancer Med. 2014; 3:702–09. 10.1002/cam4.174. 24756915 PMC4101762

[56] Chang CH, Hsiao CF, Yeh YM, Chang GC, Tsai YH, Chen YM, Huang MS, Chen HL, Li YJ, Yang PC, Chen CJ, Hsiung CA, Su WC. Circulating interleukin-6 level is a prognostic marker for survival in advanced nonsmall cell lung cancer patients treated with chemotherapy. Int J Cancer. 2013; 132:1977–85. 10.1002/ijc.27892. 23034889

[57] Wu CT, Chen MF, Chen WC, Hsieh CC. The role of IL-6 in the radiation response of prostate cancer. Radiat Oncol. 2013; 8:159. 10.1186/1748-717X-8-159. 23806095 PMC3717100

[58] Krishnamurthy S, Warner KA, Dong Z, Imai A, Nör C, Ward BB, Helman JI, Taichman RS, Bellile EL, McCauley LK, Polverini PJ, Prince ME, Wicha MS, Nör JE. Endothelial interleukin-6 defines the tumorigenic potential of primary human cancer stem cells. Stem Cells. 2014; 32:2845–57. 10.1002/stem.1793. 25078284 PMC4198458

[59] Finkel KA, Warner KA, Kerk S, Bradford CR, McLean SA, Prince ME, Zhong H, Hurt EM, Hollingsworth RE, Wicha MS, Tice DA, Nör JE. IL-6 Inhibition With MEDI5117 Decreases The Fraction of Head and Neck Cancer Stem Cells and Prevents Tumor Recurrence. Neoplasia. 2016; 18:273–81. 10.1016/j.neo.2016.03.004. 27237319 PMC4887598

[60] Toyoshima Y, Kitamura H, Xiang H, Ohno Y, Homma S, Kawamura H, Takahashi N, Kamiyama T, Tanino M, Taketomi A. IL6 Modulates the Immune Status of the Tumor Microenvironment to Facilitate Metastatic Colonization of Colorectal Cancer Cells. Cancer Immunol Res. 2019; 7:1944–57. 10.1158/2326-6066.CIR-18-0766. 31554639

[61] Ohno Y, Toyoshima Y, Yurino H, Monma N, Xiang H, Sumida K, Kaneumi S, Terada S, Hashimoto S, Ikeo K, Homma S, Kawamura H, Takahashi N, et al. Lack of interleukin-6 in the tumor microenvironment augments type-1 immunity and increases the efficacy of cancer immunotherapy. Cancer Sci. 2017; 108:1959–66. 10.1111/cas.13330. 28746799 PMC5623732

[62] Hsiao YW, Liao KW, Chung TF, Liu CH, Hsu CD, Chu RM. Interactions of host IL-6 and IFN-gamma and cancer-derived TGF-beta1 on MHC molecule expression during tumor spontaneous regression. Cancer Immunol Immunother. 2008; 57:1091–104. 10.1007/s00262-007-0446-5. 18259750 PMC11029876

